# COVID-19 and *Mycobacterium* coinfection in Brunei Darussalam: case series

**DOI:** 10.5365/wpsar.2023.14.3.1011

**Published:** 2023-08-18

**Authors:** Babu Ivan Mani, Panduru Venkata Kishore, Wai Yan Khine, Dilip Joseph Thottacherry, Pui Lin Chong, Muhamad Syafiq Abdullah, Rosmonaliza Asli, Natalie Raimiza Momin, Noor Affizan Rahman, Chee Fui Chong, Vui Heng Chong

**Affiliations:** a National Isolation Centre, Ministry of Health, Tutong, Brunei Darussalam.; b Pengiran Muda Mahkota Pengiran Muda Haji Al-Muhtadee Billah Hospital, Tutong, Brunei Darussalam.; c Raja Isteri Pengiran Anak Saleha Hospital, Bandar Seri Begawan, Brunei Darussalam.; d Suri Seri Begawan Hospital, Belait, Brunei Darussalam.

Coinfection with severe acute respiratory syndrome coronavirus 2 (SARS-CoV-2) and other respiratory pathogens is not uncommon and has been reported to be associated with less favourable outcomes. (*1*) As a consequence of disruptions to health-care services due to the coronavirus disease (COVID-19) pandemic, there have been delays in patients presenting and being diagnosed with pulmonary tuberculosis (PTB) in areas where tuberculosis (TB) is endemic. (*2*, *3*) Diagnoses of TB infection declined in 2020, ranging from 16% to 41% reduction in the nine countries with the most TB cases documented previously. (*3*) Therefore, coinfections with COVID-19 and TB are expected, and patients with coinfection have been shown to do less favourably, including patients who have already recovered from PTB. (*4*, *5*) COVID-19 infection can also increase the risk of progression to TB disease or reactivation of previous TB, either due to the immunosuppressive effects of COVID-19 or from treatment, such as the use of steroids. (*6*, *7*) Therefore, timely diagnosis of TB and COVID-19 coinfection in TB-endemic countries is important.

Brunei Darussalam is a TB-endemic country and TB remains a public health problem despite rates declining from 106/100 000 population in 2000 to 64/100 000 in 2019. The rate increased in 2020 to 82/100 000 despite

the disruption to health-care services caused by the COVID-19 pandemic. The rate dropped to a pre-pandemic level of 61/100 000 in 2021, (*8*) which was likely related to some services returning to normal. The COVID-19 outbreak in Brunei Darussalam started on 9 March 2020, and by 5 November 2021, the number of COVID-19 cases recorded was 13 673. (*9*) We report our experience with patients who were coinfected with COVID-19 and *Mycobacterium* during the second wave of COVID-19 that started on 7 August 2021.

## CASE SERIES

### COVID-19 and *Mycobacterium* coinfection

Of the 1490 adult patients admitted to the National Isolation Centre for COVID-19 infection between 7 August and 6 November 2021, during the second wave, seven were coinfected with TB, giving a coinfection rate of 4.7%. These included three new cases of *Mycobacterium* infection: two were diagnosed with PTB and COVID-19 coinfection, and one tested negative by sputum smear during admission but was later confirmed by culture to be infected with *Mycobacterium fortuitum*. The other four patients were already being treated for TB when they became infected with COVID-19: three had PTB and one had TB lymphadenitis (**Table 1**). The median age of patients with coinfection was 43.5 years (range: 15–71 years), with a male to female ratio of 4:3. Five patients had comorbidities: diabetes mellitus (*n* = 5), hypertension (*n* = 3), dyslipidaemia (*n* = 3), kidney disease (*n* = 2) and bladder cancer (*n* = 1). Four patients had not been vaccinated for COVID-19. Chest X-rays (CXRs) were abnormal in six patients. All patients tested negative for HIV.

**Table 1:** Details of patients coinfected with coronavirus disease (COVID-19) and *Mycobacterium* species, Brunei Darussalam, 7 August to 6 November 2022

Case no.	Nationality	Sex/age (years)	Medical history	Chest X-ray	Tuberculosis				COVID-19					Other coinfection	Length of stay (days)	Outcome of co- infection
					TB status	Risk	Sputum smear for AFB during hospitalization	Culture for AFB	Vaccination status	Risk	Ctv at diagnosis	Disease severity^a^	Treatment			
1	Bruneian	F/53	DM, CKD, HT, DLD, obesity	Abnormal	New PTB	None	3 negative smears; positive on line probe assay	*M. tuberculosis*	Complete	Positive contact (son)	12.7	Moderate	Casirivimab and imdevimab	None	10	Alive
2	Indian	M/45	Newly diagnosed DM	Extensive consolidations and cavities	New PTB	Treated for PTB 2 years previously	Positive	*M. tuberculosis*	Complete	None	23.6	Moderate	None	None	11	Alive
3	Bruneian	M/31	None	Abnormal	New NTM	None	Negative	*M. fortuitum*	Complete	Positive contact (brother-in-law)	37.1	Moderate	None	None	5	Alive
4	Bruneian	F/50	DM, HT, DLD	Fibrosis and cavities; diffuse ground glass opacities in all zones	PTB and already on treatment	Mother > 30 years ago	3 negative smears	*M. tuberculosis*	Unvaccinated	Positive contact (hospitalization)	17.3	Moderate	None	None	12	Alive
5	Bruneian	M/42	DM, HT, DLD, pre-ESRD	Right-middle and lower zone consolidation	PTB and already on treatment	None	2 negative smears	*M. tuberculosis*	Unvaccinated	Positive contact (hospitalization)	16.8	Severe	None; contraindicated by pre-ESRD	None	4	Started on haemodialysis; died of comorbi- dities and COVID-19
6	Bruneian	M/71	DM, stage 4 bladder cancer	Right apical fibrosis; left lower zone opacities	PTB and already on treatment	Relapse	3 negative smears	*M. tuberculosis*	Unvaccinated	Positive contact (family members)	13.5	Severe	Fondaparinux and dexamethasone	Secondary *Klebsiella pneumoniae* identified in sputum; chest infection was treated	23	Alive
7	Bruneian	F/15	None	Normal	EPTB and already on treatment	Grandfather and father	3 negative smears	*M. tuberculosis*	Ineligible	Positive contact (family member)	19.0	Mild	None	*Pseudomonas aeruginosa* (sputum): not treated	15	Alive

AFB: acid-fast bacilli; CKD: chronic kidney disease; Ctv: cycle threshold value; DLD: dyslipidaemia; DM: diabetes mellitus; EPTB: extrapulmonary tuberculosis; ESRD: end-stage renal disease; F: female; HT: hypertension; M: male; NTM: non-tuberculous mycobacteria; TB: tuberculosis.

^a^ Disease severity was categorized as mild – category 1 (asymptomatic) and category 2 (mild symptoms of COVID-19); moderate – category 3 (abnormal CXR); and severe or critical – category 4 (needed supplemental oxygen) and category 5 (needed mechanical ventilation with or without other organ failure).

### Clinical information

#### New cases of pulmonary Mycobacterium *infection*

All three newly diagnosed cases with *Mycobacterium* infection had been vaccinated against COVID-19 (two doses) and were categorized as having moderate COVID-19 based on our criteria: mild – category 1 (asymptomatic) and category 2 (mild symptoms of COVID-19); moderate – category 3 (abnormal CXR); severe or critical – category 4 (needed supplemental oxygen) and category 5 (needed mechanical ventilation with or without other organ failure). (*10*)

**Fig. 1:**
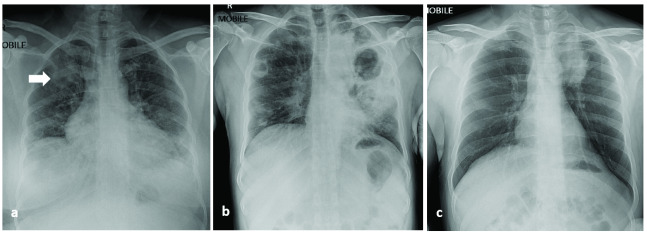
Admission chest X-ray for case 1 (a), case 2 (b) and case 3 (c)

Case 1 was a 53-year-old female with diabetes mellitus, chronic kidney disease, hypertension, dyslipidaemia and obesity who presented with chronic cough, weight loss, fever, rhinorrhoea, anorexia and dyspnoea on exertion. The admission CXR showed right upper lobe opacities (**Fig. 1a**). Sputum smear testing was negative for acid fast bacilli (AFB) but the Hain GenoType Line Probe assay (Hain Lifescience, Germany) was positive for *Mycobacterium tuberculosis*, which was confirmed by culture. This patient was treated with specific COVID-19 antibodies (casirivimab and imdevimab; Roche Pharmaceutical, Switzerland) and was also started on standard anti-TB treatment (2 months of rifampicin, isoniazid, ethambutol and pyrazinamide followed by 4 months of rifampicin and isoniazid). She reported no contact with anyone who had PTB, but she was positive for contact with COVID-19. She was categorized as having moderate COVID-19.

Case 2 was a 45-year-old male expatriate worker from India who initially presented to another hospital with mild cough and abnormal CXR (**Fig. 1b**). He tested positive for COVID-19 and was transferred to the National Isolation Centre for treatment. He did not report haemoptysis, fever or weight loss and denied any history of PTB. He was also newly diagnosed with diabetes mellitus. Interestingly, a CXR done 7 months previously during a pre-employment medical fitness check was abnormal (showing a left lung nodule). Sputum smear and culture for PTB at that time were negative. He defaulted from follow up and did not have further evaluation. During the present hospitalization, sputum smear tests were positive for PTB. On repeated inquiry, it was revealed that he had been previously treated for PTB in India 2 years earlier. He was treated as a recurrent case of PTB with standard treatment. He reported no positive contact for COVID-19. He was categorized as having moderate COVID-19.

Case 3 was a 31-year-old male without any comorbidities who presented with sore throat, new onset cough and diarrhoea. CXR showed left-medial upper zone opacity (**Fig. 1c**). Sputum smear for AFB and line probe assay were both negative. His symptoms resolved and he was discharged. Follow-up review revealed that the sputum culture was positive for *M. fortuitum*, and he was started on treatment as a case of infection with nontuberculous mycobacteria (NTM), as per our guidelines. This patient had no risk factors for NTM but had a positive contact for COVID-19. He was categorized as having moderate COVID-19.

[**insert** Figure 1]

All three cases were discharged after testing negative for SARS-CoV-2 by polymerase chain reaction (PCR), after 5 to 11 days of hospitalization.

#### *Cases with* Mycobacterium *infection already being treated*

Four patients were undergoing treatment for TB infection when they became infected with SARS-CoV-2. Three were adults with PTB (cases 4–6) and one (case 7) was an adolescent with TB lymphadenitis. All patients continued their anti-TB treatment and sputum smear testing during hospitalization until sputum smear tests were negative for AFB.

Case 4 was a 50-year-old female with diabetes mellitus, hypertension and dyslipidaemia who was admitted to another hospital with cough, dyspnoea, lethargy and reduced appetite. She was transferred to the National Isolation Centre when she tested positive for SARS-CoV-2. Her risk factor for COVID-19 was a positive contact in the ward at the previous hospital. Her CXR was abnormal, but she did not require supplemental oxygen. She did not require any specific treatment for COVID-19. She was categorized as having moderate COVID-19.

Case 5 was a 42-year-old male with diabetes mellitus, hypertension, dyslipidaemia and predialysis chronic kidney disease who presented with cough and dyspnoea. His CXR was abnormal, showing consolidation in the right-middle and lower zones. He was started on high-flow nasal oxygen and commenced dialysis. He was categorized as having severe COVID-19. However, treatment for COVID-19 was contraindicated due to his end-stage kidney disease, so it was not started. His condition was stable, but he had a sudden cardiac arrest and died 4 days after hospitalization.

Case 6 was a 71-year-old male with diabetes mellitus and stage 4 urinary bladder cancer who was admitted with exertional dyspnoea and cough. His CXR was abnormal. He was started on supplemental oxygen, low-molecular-weight heparin and dexamethasone. He was also treated with a course of antibiotics for secondary pulmonary coinfection with *Klebsiella pneumoniae*. He was categorized as having severe COVID-19. His condition improved with treatment.

Case 7 was a 15-year-old female who was being treated for lymphadenitis of the neck. She was admitted with cough, dyspnoea and chest pain. Her CXR was normal. *Pseudomonas aeruginosa* was isolated from her sputum. Because she was improving, no treatment was initiated. She was categorized as having mild COVID-19.

All surviving cases were discharged after testing negative for SARS-CoV-2 on PCR, after 12 to 23 days of hospitalization.

### Outcomes and follow up

The length of hospitalization for all seven cases ranged from 4 to 23 days (median: 11). There was one death (case 5) due to severe COVID-19 and significant comorbidities, giving a mortality rate for COVID-19 and *Mycobacterium* coinfection of 14.3%. All other patients recovered from their COVID-19 infection and completed their TB treatment. However, case 6 died from advanced cancer of the bladder 271 days after recovering from COVID-19.

## Discussion

COVID-19 is a highly infectious disease and it is not surprising that patients with other infections become infected with it. One systematic review and meta-analysis reported a high prevalence of coinfection among COVID-19 patients, with the most common coinfection being bacterial (pooled prevalence: 20.9%), followed by fungal (12.6%) and viral (12.6%). (*11*) The most common bacterial coinfections reported were primary or secondary bloodstream infections or lower respiratory tract infections. (*12*) COVID-19 and TB coinfection has been less commonly reported, and when reported it has been mainly as case reports or small case series. A systematic review of studies looking at COVID-19 and TB coinfection up to February 2021 identified 11 case series and 20 case reports with a total 146 patients, most from China and India, two of the most populous nations where TB remains endemic. (*13*) During the second wave of COVID-19 in Brunei Darussalam, we encountered seven cases of coinfection with COVID-19 and Mycobacterium, giving a coinfection rate of 4.7%.

Among our patients with coinfection, three had recently detected pulmonary mycobacteria infections, two had PTB and one had pulmonary *M. fortuitum*. All new cases had changes on CXR reflecting coinfection with pulmonary *Mycobacterium* and COVID-19. The categorization of COVID-19 as moderate for these three patients was based on their CXR changes. If CXR had not been part of routine assessment, these diagnoses of pulmonary *Mycobacterium* would have been missed. Fortunately, our management protocol required selective follow up of patients with unresolved issues or pending investigations, and this prevented us from missing the case of NTM infection. Of major concern was that one patient (case 2), an expatriate labourer, had tested negative for PTB 7 months before his admission with COVID-19. Given his CXR findings, it is quite certain he already had active PTB at that time. Unfortunately, he defaulted from his scheduled follow-up appointment and did not have any further evaluation. If he had not been evaluated during his most recent admission, the diagnosis of PTB would have been missed again, posing a risk for continued community spread.

Hospitalizations were uncomplicated and all patients were discharged within 5 to 11 days, except for case 5 who died of comorbidities 4 days after admission. Importantly, all had received two doses of COVID-19 vaccine, and this may have mitigated the impact of COVID-19.

For cases known to have PTB and already on treatment, excluding the adolescent patient who was being treated for TB lymphadenitis (case 7), COVID-19 manifestations were more severe, with two cases needing supplemental oxygen. These three cases had pre-existing pulmonary damage from PTB in addition to other significant comorbidities. In addition to changes on CXR due to PTB, there were also changes due to COVID-19, such as ground glass opacities or consolidations. Severe manifestations are not unexpected in these patients, given their already compromised pulmonary function. Furthermore, none of the patients had been vaccinated for COVID-19. The adolescent patient being treated for TB lymphadenitis was not eligible for vaccination at that time because the COVID-19 vaccine had not yet been approved for people younger than 18 years. Patients in this group were hospitalized for longer compared with cases recently diagnosed with *Mycobacterium* infection.

There was one death in our series, giving a mortality rate of 14.3%, comparable to the 13.0% reported by Koupaei et al., and compared with the 6.6% rate of deaths from COVID-19 without coinfection. (*13*) A meta-analysis reported increased disease severity and mortality among those with coinfections compared with those without TB coinfection. (*14*) A study from the United States of America reported higher mortality among cases with TB–COVID-19 coinfection: it was two times higher compared with persons with TB before the pandemic and 20 times higher compared with persons with COVID-19 alone. (*15*) This may be due to the synergistic effects of coinfections and also because patients with PTB may have damaged lungs. Furthermore, COVID-19 can progress rapidly, leading to fulminant lung damage. Therefore, early diagnosis is important, especially now that treatment for SARS-CoV-2 is available.

As the pandemic continues, and even long after the pandemic is declared over, COVID-19 will persist as an endemic illness and eventually circulate as a common respiratory viral infection. The risk of coinfections occurring with COVID-19 will persist, especially for patients with chronic diseases such as PTB. Despite our findings of only a 4.7% coinfection rate, we continue to follow our protocol to screen all PTB patients for COVID-19 and vice versa if the COVID-19 patients exhibit features on imaging or clinical features of TB, especially because treatments are available.

The main limitation of our study is the small sample size: we had only seven patients with coinfection, including a patient with NTM infection with *M. fortuitum*. However, as a result of the management strategy in the country, all patients with COVID-19 and *Mycobacterium* coinfection were isolated and treated during the study period. Therefore, our results are representative of the whole country.

## Conclusions

Our case series showed that COVID-19 and *Mycobacterium* coinfection is uncommon, with only 4.7% of patients admitted during the second wave of the COVID-19 pandemic in Brunei Darussalam being affected. It is important to be aware that new cases of pulmonary *Mycobacterium* infection may present with COVID-19 as a coinfection, and similarities in clinical manifestations may result in missed diagnoses. Similarly, patients being treated for TB are susceptible to COVID-19. Almost all of our patients had moderate to severe COVID-19 disease and, fortunately, most recovered with or without specific COVID-19 treatment. There was one death (mortality rate: 14.3%) in a patient with significant comorbidities. Even though COVID-19 and TB coinfections were uncommon, we will continue to follow our protocol to screen for COVID-19 coinfection among patients admitted with PTB, either known or newly diagnosed. Similarly, we will also continue to screen for PTB in patients admitted for COVID-19 who exhibit features of PTB, especially if their CXR shows changes suggestive of PTB.
